# iATT liver fat quantification for steatosis grading by referring to MRI proton density fat fraction: a multicenter study

**DOI:** 10.1007/s00535-024-02096-w

**Published:** 2024-03-30

**Authors:** Masashi Hirooka, Sadanobu Ogawa, Yohei Koizumi, Yuichi Yoshida, Tatsuya Goto, Satoshi Yasuda, Masahiro Yamahira, Tsutomu Tamai, Ryoko Kuromatsu, Toshihisa Matsuzaki, Tomoyuki Suehiro, Yoshihiro Kamada, Yoshio Sumida, Yoichi Hiasa, Hidenori Toyoda, Takashi Kumada

**Affiliations:** 1 Department of Gastroenterology and Metabology, Ehime University Graduate School of Medicine, Toon, Ehime 791-0295 Japan; 2 Department of Imaging Diagnosis, Ogaki Municipal Hospital, Ogaki, Japan; 3 Department of Gastroenterology and Hepatology, Suita Municipal Hospital, Suita, Japan; 4 Department of Gastroenterology, Ogaki Municipal Hospital, Ogaki, Japan; 5 Department of Clinical Laboratory Medicine, Suita Municipal Hospital, Suita, Japan; 6 Department of Gastroenterology, Kagoshima City Hospital, Kagoshima, Japan; 7 Division of Gastroenterology, Department of Medicine, Kurume University School of Medicine, Kurume, Japan; 8 Department of Gastroenterology, Sasebo City General Hospital, Sasebo, Nagasaki Japan; 9 Clinical Research Center, National Hospital Organization Nagasaki Medical Center, Omura, Nagasaki Japan; 10 Department of Advanced Metabolic Hepatology, Graduate School of Medicine, Osaka University, Osaka, Japan; 11 Graduate School of Healthcare Management, International University of Healthcare and Welfare, Tokyo, Japan; 12 Department of Nursing, Faculty of Nursing, Gifu Kyoritsu University, Ogaki, Japan

**Keywords:** Ultrasound, B-mode, Attenuation coefficient, Diagnosis, Steatosis

## Abstract

**Background:**

Several preliminary reports have suggested the utility of ultrasound attenuation coefficient measurements based on B-mode ultrasound, such as iATT, for diagnosing steatotic liver disease. Nonetheless, evidence supporting such utility is lacking. This prospective study aimed to investigate whether iATT is highly concordant with magnetic resonance imaging (MRI)-based proton density fat fraction (MRI-PDFF) and could well distinguish between steatosis grades.

**Methods:**

A cohort of 846 individuals underwent both iATT and MRI-PDFF assessments. Steatosis grade was defined as grade 0 with MRI-PDFF < 5.2%, grade 1 with 5.2% MRI-PDFF < 11.3%, grade 2 with 11.3% MRI-PDFF < 17.1%, and grade 3 with MRI-PDFF of 17.1%. The reproducibility of iATT and MRI-PDFF was evaluated using the Bland–Altman analysis and intraclass correlation coefficients, whereas the diagnostic performance of each steatosis grade was examined using receiver operating characteristic analysis.

**Results:**

The Bland–Altman analysis indicated excellent reproducibility with minimal fixed bias between iATT and MRI-PDFF. The area under the curve for distinguishing steatosis grades 1, 2, and 3 were 0.887, 0.882, and 0.867, respectively. A skin-to-capsula distance of ≥ 25 mm was identified as the only significant factor causing the discrepancy. No interaction between MRI-logPDFF and MRE-LSM on iATT values was observed.

**Conclusions:**

Compared to MRI-PDFF, iATT showed excellent diagnostic accuracy in grading steatosis. iATT could be used as a diagnostic tool instead of MRI in clinical practice and trials.

*Trial registration* This study was registered in the UMIN Clinical Trials Registry (UMIN000047411).

**Supplementary Information:**

The online version contains supplementary material available at 10.1007/s00535-024-02096-w.

## Introduction

Over recent years, the prevalence of steatotic liver disease has substantially increased worldwide, and the prevalence of metabolic dysfunction-associated steatotic liver disease (MASLD) has become a pressing issue affecting populations across different regions [1, 2]. Recent estimates suggest that MASLD affects approximately 30% of the populations in North America and Australasia and 25% in Western Europe [1]. The prevalence of MASLD has also been on the rise in Asia despite being historically known for its low prevalence of obesity, partially because of changing dietary habits and sedentary lifestyles. For instance, in Japan, the prevalence of MASLD has increased to approximately 20–30% and is approaching levels observed in Western countries [3, 4]. Such an increase underscores the fact that the impact of MASLD is not limited to specific geographical regions, but is a global phenomenon. Furthermore, these prevalence rates emphasize the need for robust diagnostic approaches to tackle this growing health challenge.

MASLD encompasses a spectrum of liver diseases characterized by excessive fat accumulation in hepatocytes, ranging from simple steatosis to non-alcoholic steatohepatitis, and can potentially progress to advanced fibrosis, cirrhosis, liver failure and hepatocellular carcinoma [5–7]. While the prognosis of patients with MASLD has been reported to be predominantly associated with the extent of liver fibrosis [8–12], emerging evidence has challenged this perspective, highlighting the critical role of hepatic fat content as a prognostic indicator [13]. Thus far, the severity of MASLD is determined by the degree of liver fibrosis without taking the potential impact of simple steatosis into account. However, even at the stage of simple steatosis, patients could have worse outcomes than healthy people, as shown by a recent study from Sweden [13]. Furthermore, previous studies reported that advanced steatotic liver disease could be linked to accelerated fibrogenesis, exacerbating the risk of disease progression [14] and that reducing the liver fat content could improve liver fibrosis [15].

Consequently, liver fat content quantification has received considerable clinical interest. In the current landscape of liver fat quantification, the traditional reliance on liver biopsy, which is prone to sampling error and is associated with high-risk complications, has been gradually replaced with magnetic resonance imaging (MRI)-based proton density fat fraction (MRI-PDFF), a non-invasive approach that offers improved accuracy and provides valuable insights [16, 17]. Nonetheless, the application of MRI-PDFF is not devoid of limitations. MRI-PDFF relies on MRI, thereby posing limitations in patients with metal implants within their bodies. Additionally, MRI-PDFF often takes a relatively long time to complete, which can be particularly inconvenient for patients with limited mobility. It also utilizes advanced medical imaging technology, which can result in high examination costs, thus limiting its accessibility in certain regions or facilities. Although a test for diagnosing hepatic steatosis should expectedly be used from the screening stage, these factors make the use of MRI-PDFF impractical during the screening stage.

Amid these challenges, the utilization of B-mode ultrasound attenuation-based methods is emerging as a promising alternative. Various non-invasive techniques for hepatic fat content assessment, including B-mode ultrasound-based methods with the ultrasound-guided attenuation parameter (UGAP) [18, 19], ATI [20, 21], and ATT [22–24] have been proposed. Among these, only UGAP has been validated in a large-scale multicenter study involving over 1000 cases; Imajo et al. showed that UGAP was comparable to MRI-PDFF in terms of being a diagnostic tool for hepatic steatosis [18]. Hence, clinical trials are crucial for validating the high diagnostic performance of other tests investigated by large multicenter studies.

The present prospective multicenter study aimed to investigate the diagnostic accuracy of grading steatosis with reference to MRI-PDFF in a large cohort.

## Methods

This prospective study involved multi-institutional cohorts in Japan. Online Resource 1 shows the study protocol. Patients were recruited from seven liver centers throughout Japan—namely, Ehime University Hospital, Ogaki Municipal Hospital, Suita Municipal Hospital, Kagoshima City Hospital, Kurume University Hospital, Sasebo City General Hospital, and National Hospital Organization Nagasaki Medical Center. A total of 846 patients with chronic liver disease who underwent MRI-PDFF and iATT from May 2021 to March 2023 were enrolled. However, patients (i) who were unable to hold their breath for several seconds for iATT measurement; (ii) who might experience difficulties in undergoing MRI owing to claustrophobia, presence of magnetic material within their bodies, tattoos, implanted pacemakers, etc.; or (iii) who did not consent to study participation were excluded from the analysis.

This study was conducted in accordance with the ethical principles outlined in the 2013 Declaration of Helsinki and the 2018 Declaration of Istanbul and was registered in the UMIN Clinical Trials Registry (UMIN000047411).

### iATT measurements

**Fig. 1:**
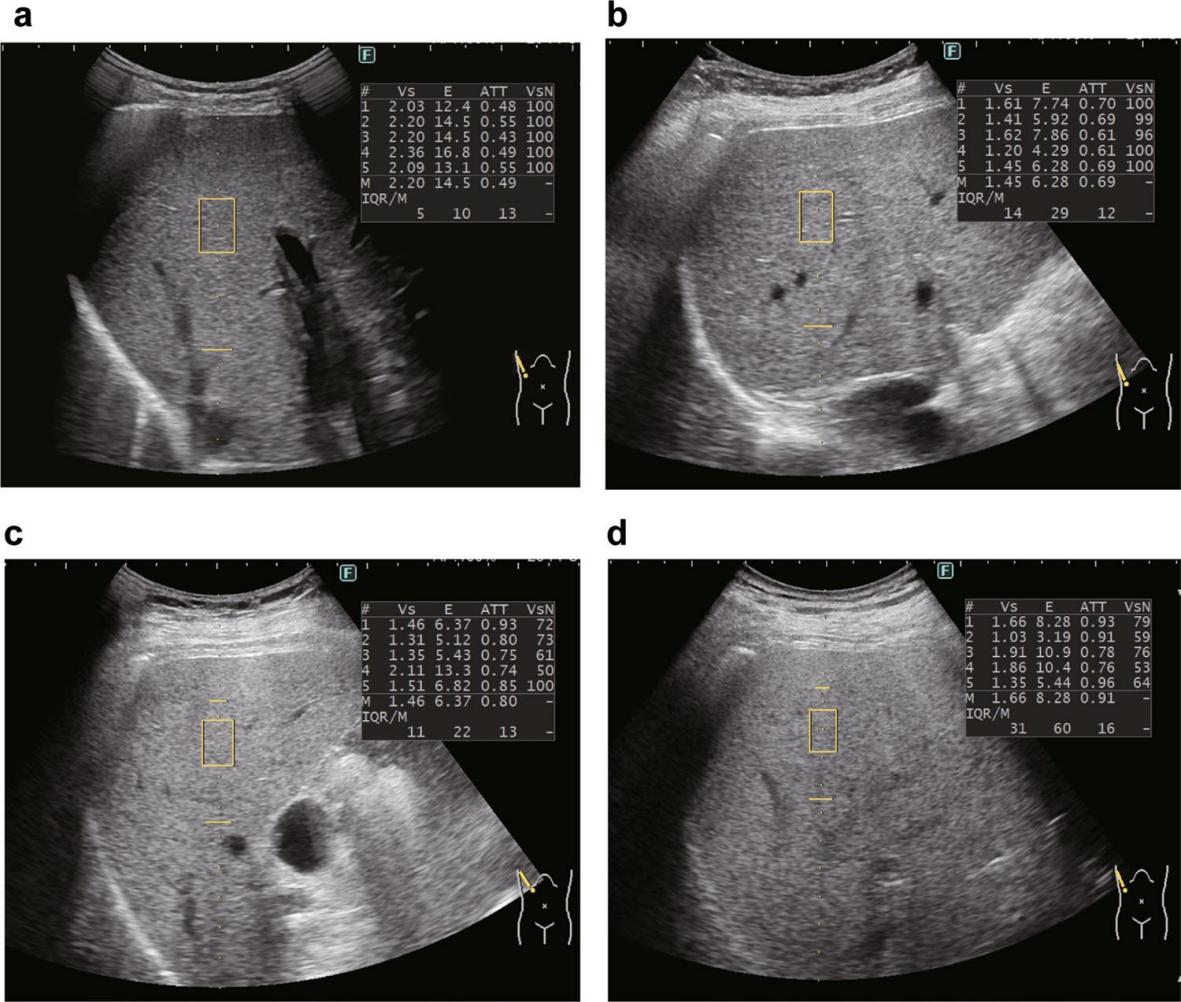
Measurement of attenuation coefficient with iATT. Ultrasound images are presented for each S grade defined by MRI-PDFF. **a** Steatosis grade 0 (iATT value, 0.49; IQR/M, 13%; minimum value of VsN, 100), **b** steatosis grade 1 (iATT value, 0.69; IQR/M, 12%; minimum value of VsN, 96), **c** steatosis grade 2 (iATT value, 0.80; IQR/M, 13%; minimum value of VsN, 50), **d** steatosis grade 3 (iATT value, 0.91; IQR/M, 16%; minimum value of VsN, 53). *IQR/M* interquartile range/median, *MRI-PDFF* magnetic resonance imaging-proton density fat fraction

Examinations were performed using the ARIETTA 850 US system (Fujifilm Healthcare, Tokyo, Japan) with a convex broadband probe. Measurements were obtained from the right intercostal space while the patients held their breath. Considering that the attenuation coefficient could be obtained together with liver stiffness, the region of interest in the liver was placed perpendicular to the liver capsule [25–27], and the region of interest was set in the homogenous liver parenchyma without any vessel (Fig. 1). The manufacturer’s quality standard for iATT was defined as VsN > 50% and interquartile range/median (interquartile range/median) ratios < 30%; thus, measurements were repeated until five valid measurements resulting in both VsN > 50% and interquartile range/median < 30% were obtained [28]. The median value for fat quantification (expressed as decibel/meter/megahertz [dB/m/MHz]) was used for statistical analysis.

### MRI-PDFF

MRI was performed using Discovery MR750w 3.0 T system (GE Healthcare, Waukesha, WI, USA). PDFF was measured using the multi-echo Dixon method (IDEAL-IQ sequence), as previously described [18]. MRI-PDFF was measured with a single region of interest (20 × 20 × 20 mm^3^) placed in liver segment V, VI, VII, or VIII and was subsequently analyzed by hepatologists who were blinded to the iATT results at each institution (MRI interpretations with > 3 years of experience). Steatosis grade was defined as grade 0 with MRI-PDFF < 5.2%, grade 1 with 5.2% MRI-PDFF < 11.3%, grade 2 with 11.3% MRI-PDFF < 17.1%, and grade 3 with MRI-PDFF of 17.1% [16]. Out of 846 patients, 566 underwent magnetic resonance elastography (MRE). Thus, the fibrosis stage based on MRE was defined as follows: F0, < 2.5 kPa; F1, 2.5–3.3 kPa; F2, 3.4–4.7 kPa; and ≥ F3, ≥ 4.8 kPa [16].

### Statistical analysis

Continuous variables are expressed as median (interquartile range [IQR]). The relationship between MRI-logPDFF and iATT was examined using Pearson’s correlation coefficient (*r*), which was interpreted as follows: |*r*| < 0.2, minimal; |*r*| = 0.2–0.4, weak; |*r*| = 0.4–0.7, moderate; and |*r*| = 0.7, strong. Bias, defined as the average difference between MRI-PDFF and iATT measurements, was assessed by conducting a Bland–Altman analysis [29–32]. In Bland–Altman analysis, data provide a distribution histogram of the differences, which should be normally distributed. Moreover, if necessary, data transformation is recommended [33]. Therefore, we visualized whether the data were normally distributed using a quantile–quantile plot, and if they were not, we performed a logarithmic transformation. The 95% limits of agreement (LOA) were calculated and shown in Bland–Altman plots. The correlation between the difference and mean of measurements, fixed error, and proportional error was also calculated. The proportional error depended on the number of cases; specifically, when the proportional error was significant, it was defined as “no proportional error” if the following criteria for assessing the compatibility of results of two modalities were defined: (i) the percentage LOA was less than the expected LOA and (ii) > 75% of the difference was less than the expected LOA. The cases for which the Bland–Altman plots illustrated values that did not fall between the upper and lower LOA lines were considered to be divergent.

Based on the 95% confidence interval (CI) of the intraclass correlation coefficient estimate, values < 0.5, 0.5–0.75, 0.75–0.9, and > 0.90 were considered to indicate poor, moderate, good, and excellent reliability, respectively [34, 35]. In the Bland–Altman plots, dots not within the bias (± 1.96 standard deviation) were defined as divergence, and the causes of divergence were analyzed by regression analysis. The accuracy of iATT was assessed by determining the sensitivity and specificity for each value of each test and by constructing receiver operating characteristic curves to plot sensitivity against (1 − specificity) for each value. Additionally, the area under the receiver operating characteristic curve, the most commonly used index of accuracy, was calculated, with the area under curve values close to 1.0 indicating high diagnostic accuracy. The sensitivity, specificity, positive predictive value, negative predictive value, positive likelihood ratio, and negative likelihood ratio were calculated to evaluate the overall accuracy of iATT. Optimal cutoff values were selected to maximize the sum of sensitivity and specificity of the Youden index, and cutoff values with at least 90% sensitivity and specificity were individually selected. The required sample size was calculated for a suitable receiver operating characteristic analysis. To achieve the proportion of cases with steatosis grades 1, 2, and 3 of 0.5, 0.3, and 0.1, respectively, and the area under the receiver operating characteristic curve of 0.8 and the required number of cases was calculated to be 194, 272, and 730, respectively.

An interaction between iATT and fibrosis was confirmed via a comparison of iATT values for each F stage defined by MRE in each S grade determined by MRI-PDFF. The association between liver fibrosis stage and iATT was evaluated by conducting the Jonckheere–Terpstra analysis. Multiple comparisons were performed using the Steel–Dwass test. Statistical analyses were performed using STATA version 15.0 (StataCorp, College Station, Texas, USA), with statistical significance set at *p* < 0.05.

## Results

### Study population

A total of 846 patients were enrolled in this study after excluding 13 patients (VsN < 50%, 2; interquartile range/median > 30%, 10) (Online Resource 1). Table 1 summarizes the characteristics and laboratory data of the enrolled patients. The median body mass index was 24.8 kg/m^2^ (IQR, 22.1–28.1 kg/m^2^). The median skin-to-capsula distance (SCD) was 18 mm (IQR, 15–20 mm).

**Table 1:** Patient characteristics (*n* = 846)

Characteristics	
Age (years)	65 (53–73)
Male sex (*n*, %)	439 (51.9%)
BMI (kg/m^2^)	24.8 (22.1–28.1)
SCD (mm)	18 (15–20)
AST (U/L)	30 (22–50)
ALT (U/L)	30 (18–56)
GGT (U/L)	43 (23–103)
Platelet count (× 10^4^/μL)	20.3 (14.7–25.2)
HbA1c (%)	5.8 (5.5–6.4)
Etiology SLD/ALD/HBV/HCV/AIH/PBC/others	337: 44: 155: 117: 38: 39: 116
iATT (dB/cm/MHz)	0.69 (0.59–0.78)
MRI-PDFF (%)	5.72 (2.62–12.29)

Data are expressed as median and interquartile range or as *n* (%)

*AIH* autoimmune hepatitis, *ALD* alcoholic liver disease, *ALT* alanine aminotransferase, *AST* aspartate aminotransferase, *BMI* body mass index, *GGT* g-glutamyl transferase, *HbA1c* hemoglobin A1c, *HBV* hepatitis B virus antigen-positive, *HCV* anti-hepatitis C virus-positive, *MRI-PDFF* magnetic resonance imaging-proton density fat fraction, *PBC* primary biliary cholangitis, *SCD* skin-to-capsula distance, *SLD* steatotic liver disease

Online Resource 2 presents the median iATT value, as compared with the steatosis grade defined by MRI-PDFF: S0, 0.59 dB/cm/MHz (IQR, 0.54–0.65 dB/cm/MHz); S1, 0.71 dB/cm/MHz (IQR, 0.67–0.77 dB/cm/MHz); S2, 0.78 dB/cm/MHz (IQR, 0.74–0.83 dB/cm/MHz); and S3, 0.82 dB/cm/MHz (IQR, 0.76–0.89). The Jonckheere–Terpstra analysis revealed that the median iATT value increased in a stepwise manner at each S grade (*p* < 0.0001). The median duration of iATT and MRI-PDFF was 18 days (IQR, 0–60 days).

### Normal probability plot of MRI-PDFF and iATT values

The normal probability of MRI-PDFF and iATT values was tested using the normal probability plot. The results indicated that iATT values were normally distributed, whereas MRI-PDFF values were not (Online Resource 3). Therefore, MRI-PDFF values were log-transformed to achieve a normal distribution (MRI-logPDFF), as in a previous study [18] (Online Resource 3).

### Reproducibility between iATT and MRI-PDFF

**Fig. 2:**
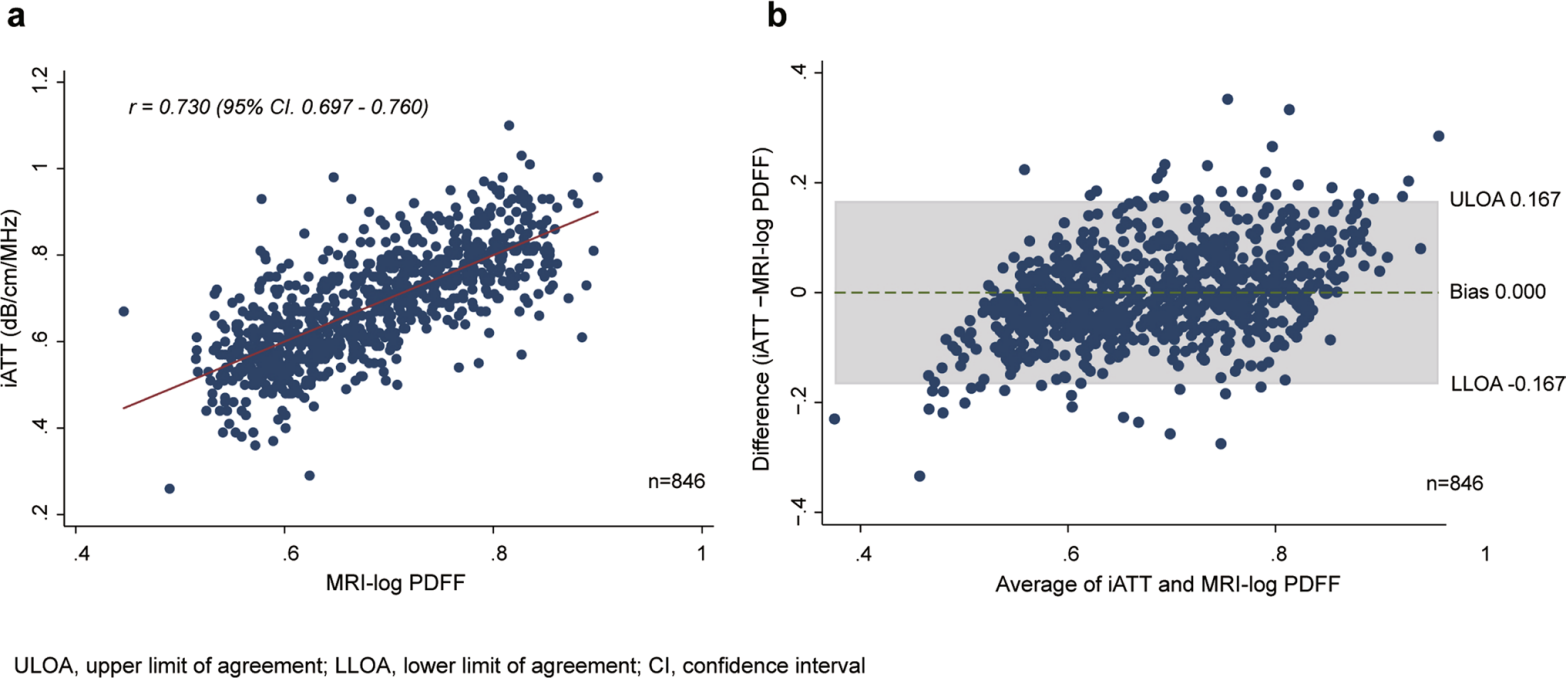
Scatterplots and Bland–Altman plots. Scatterplots and Bland–Altman plots between iATT and MRI-PDFF. **a** iATT was significantly correlated with MRI-logPDFF (*r* = 0.730, *p* < 0.0001). **b** Bland–Altman plots did not show fixed bias (0.000%). *MRI-PDFF* magnetic resonance imaging-proton density fat fraction

Linearity and systematic bias between MRI-logPDFF and iATT measurements were analyzed. As shown in Fig. 2A, the relationship between MRI-logPDFF and iATT was significantly linear (*r* = 0.730, *p* = 0.0001). The Bland–Altman plot indicated a narrow 95% LOA between iATT and MRI-logPDFF (upper LOA, 0.167; lower LOA, − 0.167; Fig. 2B). The fixed error was 0.000. The percentage error was 24.86, which was below the expected LOA of 28.98. The frequency of percentage error below the expected LOA was 91.8% (793/864). The inter-operator reliability showed a good intraclass correlation coefficient of 0.70 (95% CI 0.66–0.73). The Bland–Altman plots without values between the upper and lower LOA lines were considered to be divergent (48 cases, 5.7%; Fig. 2B). Cases above the upper LOA were considered to be overestimations of MRI-logPDFF or underestimations of MRI-logPDFF, whereas cases below the lower LOA were considered to be the opposite case. For each case above and below the upper LOA, univariate and multivariate regression analyses were performed to identify the cause; as a result, only SCD ≥ 25 mm was identified as a significant factor (Table 2). No significant difference was found with regard to the presence or absence of steatotic liver disease.

**Table 2:** Predictors of divergency between iATT and MRI-PDFF

	Odds ratio (95% CI)	*p*-value	Odds ratio (95% CI)	*p*-value
Male sex	0.845 (0.471–1.513)	0.571	0.880 (0.488–1.587)	0.672
Age ≥ 65 years	0.878 (0.490–1.572)	0.661	1.077 (0.563–2.060)	0.937
BMI ≥ 25 kg/m^2^	0.876 (0.488–1.572)	0.658		
BMI ≥ 30 kg/m^2^	1.434 (0.714–2.882)	0.311	0.815 (0.288–2.306)	0.699
SCD ≥ 20 mm	1.445 (0.795–2.626)	0.228		
SCD ≥ 25 mm	2.614 (1.251–5.461)	0.011	3.753 (1.276–11.035)	0.016
SCD ≥ 30 mm	4.242 (1.656–10.864)	0.003		
SLD vs. non-SLD	0.989 (0.545–1.794)	0.971	1.153 (0.996–1.334)	0.066
≥ S1	0.720 (0.402–1.293)	0.272		
≥ S2	1.117 (0.588–2.121)	0.736	1.267 (0.609–2.637)	0.527
≥ S3	1.385 (0.653–2.935)	0.396		
ALT ≥ 100 IU/L	0.926 (0.358–2.400)	0.875	0.887 (0.334–2.360)	0.811
Virus vs. non-virus	0.707 (0.352–1.421)	0.330		

Steatosis grade was defined as S0 with MRI-PDFF < 5.2%, S1 with 5.2% MRI-PDFF < 11.3%, S2 with 11.3% MRI-PDFF < 17.1%, and S3 with MRI-PDFF of 17.1%

Virus was defined as HBs antigen or anti-HCV positive

*ALT* alanine aminotransferase, *BMI* body mass index, *CI* confidence interval, *SCD* skin-to-capsula distance, *SLD* steatotic liver disease

### Diagnostic accuracy and cutoff value of iATT with MRI-PDFF as the reference technique

**Fig. 3:**
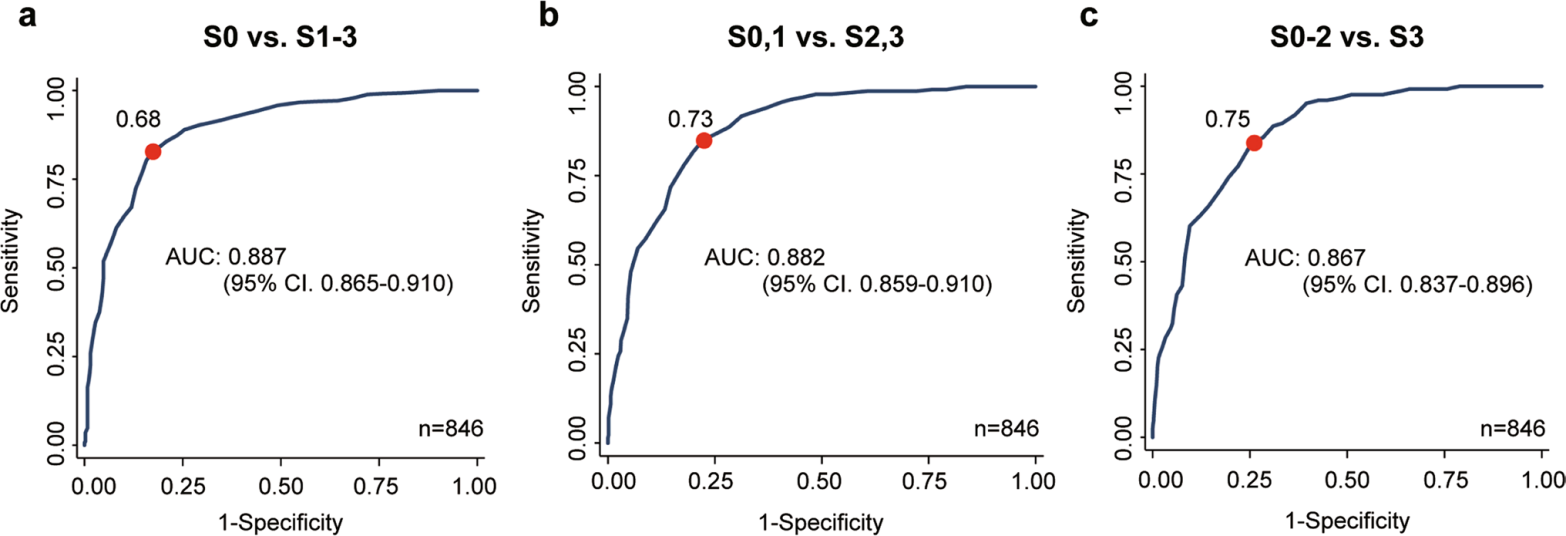
Receiver operating characteristic analysis for each steatosis grade. **a** Steatosis grade ≥ 1, **b** steatosis grade ≥ 2, **c** steatosis grade ≥ 3

The area under the curve of iATT for diagnosing S1, S2, or S3 were 0.887, 0.882, or 0.867, respectively (Fig. 3). Table 3 presents the diagnostic values. According to the Youden index, the cutoff value for the S1 grade by iATT was 0.68 dB/cm/MHz, with a sensitivity of 82.6% and specificity of 82.7%. For iATT, the cutoff values for a sensitivity of ≥ 0.90 and specificity of ≥ 0.90 were 0.64 and 0.74, respectively.

**Table 3:** Diagnostic performance of iATT for hepatic steatosis

Cutoff	Se	Sp	PPV	NPV	LR+	LR−
S1						
0.68^a^	82.6	82.7	84.6	80.4	4.77	0.21
0.64^b^	90.3	70.7	78.1	86.3	3.09	0.14
0.74^c^	61.4	91.9	89.7	67.4	7.54	0.42
S2						
0.73^a^	85.2	77.6	58.6	93.4	3.81	0.19
0.70^b^	91.7	68.7	52.1	95.7	2.93	0.12
0.79^c^	57.2	91.2	70.8	85.2	6.54	0.47
S3						
0.75^a^	83.7	74.6	35.9	96.4	3.29	0.22
0.71^b^	91.9	63.3	29.9	97.9	2.51	0.13
0.81^c^	60.2	90.5	51.7	93.0	6.30	0.44

LR−: negative likelihood ratio; LR+: positive likelihood ratio; NPV: negative predictive value; PPV: positive predictive value; Se: sensitivity; Sp: specificity

^a^Cutoff by the Youden index

^b^Cutoff for sensitivity ≥ 0.90

^c^Cutoff for specificity ≥ 0.90

According to the Youden index, the cutoff value for the S2 grade by iATT was 0.73 dB/cm/MHz, with a sensitivity of 85.2% and specificity of 77.6%. For iATT, the cutoff values for a sensitivity of ≥ 0.90 and specificity of ≥ 0.90 were 0.70 and 0.79, respectively.

According to the Youden index, the cutoff value for the S3 grade by iATT was 0.75 dB/cm/MHz, with a sensitivity of 83.7% and specificity of 74.6%. For iATT, the cutoff values for a sensitivity of ≥ 0.90 and specificity of ≥ 0.90 were 0.75 and 0.81, respectively.

**Fig. 4:**
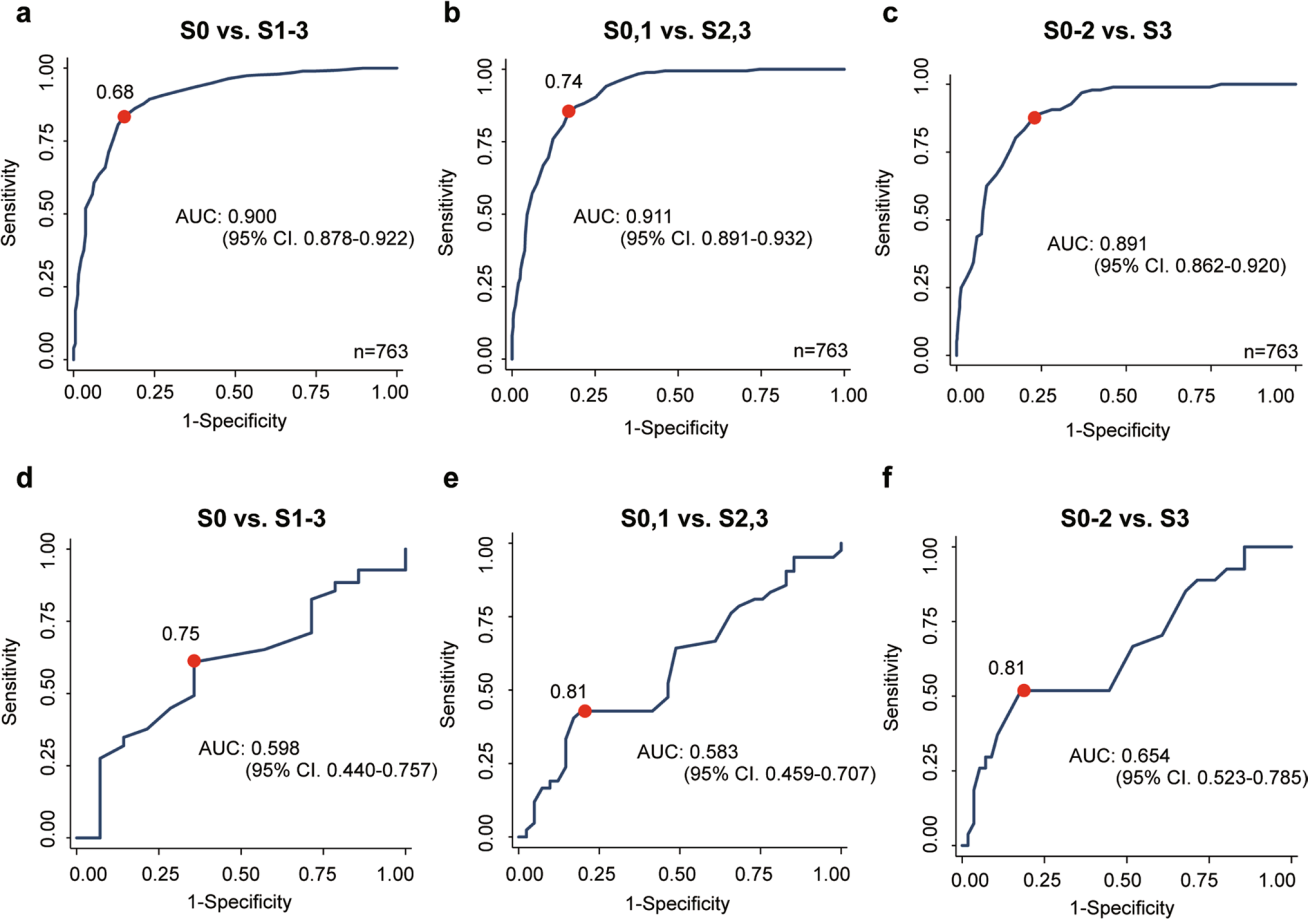
Receiver operating characteristic analysis for each steatosis grade for SCD 25-mm subdivision. **a** Steatosis grade ≥ 1 and SCD < 25 mm, **b** steatosis grade ≥ 2 and SCD < 25 mm, **c** steatosis grade ≥ 3 and SCD < 25 mm, **d** steatosis grade ≥ 1 and SCD ≥ 25 mm, **e** steatosis grade ≥ 2 and SCD ≥ 25 mm, **f** steatosis grade ≥ 3 and SCD ≥ 25 mm. *SCD* skin-to-scapula distance

Because SCD > 25 mm was extracted as a significant factor in discrepant cases, diagnostic performance was analyzed separately for patients with SCD ≥ 25 mm and those with SCD < 25 mm. Patients with SCD ≥ 25 mm had a lower area under curve, whereas those with SCD < 25 mm had an improved area under curve (Fig. 4).

### Interaction assessment

For each S grade, we investigated whether iATT values varied between different F grades. Multiple comparisons revealed no significant differences in any combinations (Online Resource 4). Furthermore, the assessment of the effect of the interaction between MRI-logPDFF and MRE-liver stiffness measurement (LSM) on iATT values with multiple regression indicated *p*-values of < 0.001 for MRI-logPDFF, 0.817 for MRE-LSM, and 0.774 for MRI-logPDFF × MRE.

## Discussion

This multicenter prospective study revealed that iATT showed excellent diagnostic performance in detecting steatotic liver disease, as well as high reproducibility when compared with MRI-PDFF, suggesting its potential as an alternative to MRI-PDFF.

The controlled attenuation parameter, which could be obtained together with the LSM using FibroScan, measures the attenuation of the ultrasound beam. The controlled attenuation parameter is an easy-to-measure tool that has been available for more than a decade, and several studies have assessed its value in liver fat content quantification, with some meta-analyses confirming its usefulness [34–36]. However, the lack of a B-mode display and the measurement discrepancies between the M probe and XL probe continue to be challenges [37, 38]. Furthermore, as reported by the recent position paper on liver fat quantification by the World Federation for Ultrasound in Medicine and Biology and the AIUM-RSNA QIBA (The American Institute of Ultrasound in Medicine-RSNA Quantitative Imaging Biomarkers Alliance) initiative article, the controlled attenuation parameter is not an adequate reference standard for evaluating the accuracy of emerging ultrasound techniques for fat quantification with attenuation coefficients [38, 39].

In the last few years, several ultrasound manufacturers have developed software for liver fat content quantification using attenuation coefficients [18–20, 23, 24]. Among them, iATT, an upgraded version of the ATT algorithm released by Fujifilm Healthcare, Tokyo, Japan (previously Hitachi Ltd., Tokyo, Japan), was introduced a few years ago [24]. Attenuation coefficient methods based on B-mode imaging, such as iATT, are excellent for hepatic steatosis assessment because they enable the confirmation of measurement sites by B-mode imaging. There is no need to purchase additional equipment for FibroScan, and most existing echo machines in the facility can be utilized for attenuation coefficient measurement through software updates. While there have been numerous high-quality reports on the controlled attenuation parameter, to the best of the authors’ knowledge, evidence from multicenter prospective studies on B-mode-based attenuation measurement methods has only been established for UGAP. Prospective multicenter studies, such as the present study, which compared iATT to MRI-PDFF, should provide a high level of evidence for other B-mode methods.

Currently, MRI-PDFF is becoming an established alternative to biopsy. Similar to UGAP, it is important to investigate the compatibility of iATT with MRI-PDFF. In addition, liver fat content quantification has been reported to have inter-operator variability in reproducibility [40]. We believed that by adjusting our research methodology to that of Imajo et al., we could confirm whether similar results to UGAP could be achieved. In many cases in this study, liver biopsy was not available to assess steatosis. As noted above, the correlation between MRI-PDFF and histological steatosis grade decreases with increasing steatosis grade; however, overall, the correlation is at an acceptable level [41]. In patients with any degree of steatosis, the relationship between PDFF and histology was predominantly linear (*r* = 0.85 [95% CI 0.80–0.89]). The American Association for the Study of Liver Diseases guidelines also indicate that liver biopsy should be considered in patients with MASLD who are at increased risk of steatohepatitis and advanced fibrosis [42]. Recently, many multicenter clinical trials have used MRI-PDFF as the reference standard; furthermore, the number of trials using biopsy tissue as the reference standard is expected to decrease owing to ethical reasons [16, 18, 43]. Therefore, in the present study, MRI-PDFF instead of histology was used as the reference standard based on the previous study.

In this study, the agreement between iATT and MRI-PDFF was evaluated by performing a Bland–Altman analysis. There was almost no fixed error. In comparison, in a large-scale study, proportional errors are more likely to occur. Therefore, as in previous studies, we established two evaluation criteria and analyzed the agreement between the two modalities [18, 44]. As both criteria were met, we concluded through the Bland–Altman analysis that iATT was compatible with MRI-PDFF. High concordance was also confirmed in the intraclass correlation analysis. Therefore, iATT could serve as an alternative to MRI-PDFF.

Subsequently, diagnostic performance was analyzed. MRI-PDFF showed a strong correlation with histological fat deposition from the early stages of S grade. By determining S1, a clear distinction can be made between healthy individuals and patients with early-stage steatotic liver disease. The use of iATT calculated by the Youden index in this study yielded favorable diagnostic performance parameters, including sensitivity, specificity, positive predictive value, and negative predictive value. A previous study in Sweden showed that even simple steatotic liver disease was associated with a worse prognosis, as compared to that in healthy individuals [13]. If the cutoff value that generates a sensitivity of 90% or higher is < 0.64, the individuals are very likely to be healthy; therefore, no therapeutic intervention is considered necessary. Conversely, if the cutoff value that yields a specificity of > 90% is > 0.74, the individuals are conceivably patients with steatotic liver disease requiring therapeutic interventions to improve prognosis. The 0.74 value, which is close to the cutoff values for S2 and S3 determined by the Youden index, supports the notion that therapeutic interventions are warranted. In a meta-analysis conducted by the authors, the ATT, before it was improved to iATT, had the disadvantage of lower sensitivity than other ultrasound attenuation methods [45]. The reason for the lower diagnostic performance of the ATT is the use of the dual-frequency method wherein two frequencies are transmitted and received simultaneously as the algorithm for evaluating attenuation coefficients. The attenuation coefficient is calculated from the slope of the signal amplitude ratio curve between intervals 40 mm and 100 mm. Meanwhile, the iATT uses the reference method, as do the UGAP and the ATT. Moreover, the attenuation coefficient is calculated from the slope of the signal ratio curve between intervals 35 mm and 75 mm to acquire reproducible data [46]. The improved diagnostic performance of iATT in comparison to that of ATT was recently reported by Ogawa et al. [46]. The correlation coefficients between iATT or ATT values and MRI‐PDFF values were 0.803 and 0.533 (*p* < 0.001). For the detection of hepatic steatosis of ≥ S1, ≥ S2, and ≥ S3, iATT had significantly higher AUROCs than ATT (*p* < 0.001, *p* < 0.001, *p* = 0.001), as demonstrated in their free downloadable graphical abstract. In this study, iATT also showed good sensitivity and demonstrated high diagnostic performance in quantifying intrahepatic steatosis, similar to other B-mode diagnostic methods.

When the causes of discrepancy between iATT and MRI-PDFF in this study were examined, SCD was the only factor identified. The measurement site for iATT is fixed at a depth of 3.5–7 cm from the body surface, unlike other testing methods. Therefore, a discrepancy between iATT and MRI might have occurred in cases with thicker subcutaneous adipose tissue. Ultrasound attenuation might be affected by the tissue between the liver and skin.

Whether ultrasound attenuation measurements are influenced by the amount of liver fibrosis remains controversial. ATT has been reported to be unaffected by fibrosis or inflammation; nonetheless, analytical methods are considered inadequate [22]. In this study, a large number of cases were examined for interactions using multiple comparisons and multiple regression analysis, and iATT was not found to be influenced by the degree of fibrosis, similar to UGAP.

The present study has some limitations. First, this study was conducted in Japan only. While racial groups are believed to exhibit no differences, an international collaborative study is required. Second, the cause of divergent cases might not have been adequately searched. Hence, we should continue to search for factors other than SCD, as almost all divergent cases do not have an SCD of 25 or higher. Third, as grade S increased, the cutoff value for iATT exhibited a stepwise increase, whereas the cutoff values for grades 2 and 3 displayed minimal differences. There are two possible reasons for this phenomenon: (i) as the degree of fat deposition increases, MRI-PDFF is not well correlated to histological steatosis grade, as compared to cases with lower fat deposition, making accurate validation more difficult in severe steatosis; and (ii) it is possible that reliance on ultrasound attenuation coefficients alone may have limitations in differentiating between grades 2 and 3. While the clinical significance of differentiating between grades 2 and 3 may be limited, if differentiation is deemed necessary, future efforts should focus on developing measurement techniques that incorporate additional ultrasound signals, such as the backscatter coefficient. Fourth, the median interval between the iATT and MRI-PDFF was slightly longer. However, we confirmed that between the two tests the participants’ weight change was not more than 5%; moreover, there was no change in their physical condition, no heavy alcohol consumption, and no history of drug treatment that might have improved their fatty liver. Ogawa et al.’s series showed no significant difference in the correlation of these parameters between iATT and MRI‐derived PDFF measurements at different intervals within 3 months [46]. In our study, the interval was no longer than 3 months.

In conclusion, iATT is highly compatible with PDFF and can be enforced as an alternative modality to PDFF. Any steatosis grade shows good diagnostic performance.

### Supplementary Information

Below is the link to the electronic supplementary material.

Supplementary file 1—Study design (TIF 4211 KB)

Supplementary file 2—iATT values according to steatosis grade. **a** grade 0, **b** grade 1, **c** grade 2, **d** grade 3 (JPG 476 KB)

Supplementary file 3—Normal probability plot (**a**, **d**). iATT values were distributed normally, whereas MRI-PDFF values were not (**b**, **e**). MRI-PDFF values were log-transformed to have a normal distribution (MRI-logPDFF; **c**, **f**). MRI-PDFF, magnetic resonance imaging-proton density fat fraction (JPG 248 KB)

Supplementary file 4—iATT values according to fibrosis stage for steatosis grade subdivision. **a** S0, **b** S1, **c** S2, **d** S3 (JPG 1603 KB)
