# Supplementary figures and images for: iATT liver fat quantification for steatosis grading by referring to MRI proton density fat fraction: a multicenter study
Source: J Gastroenterol. 2024 Mar 30;59(6):504–14. doi: 10.1007/s00535-024-02096-w (PMC11128405; doi:10.1007/s00535-024-02096-w)

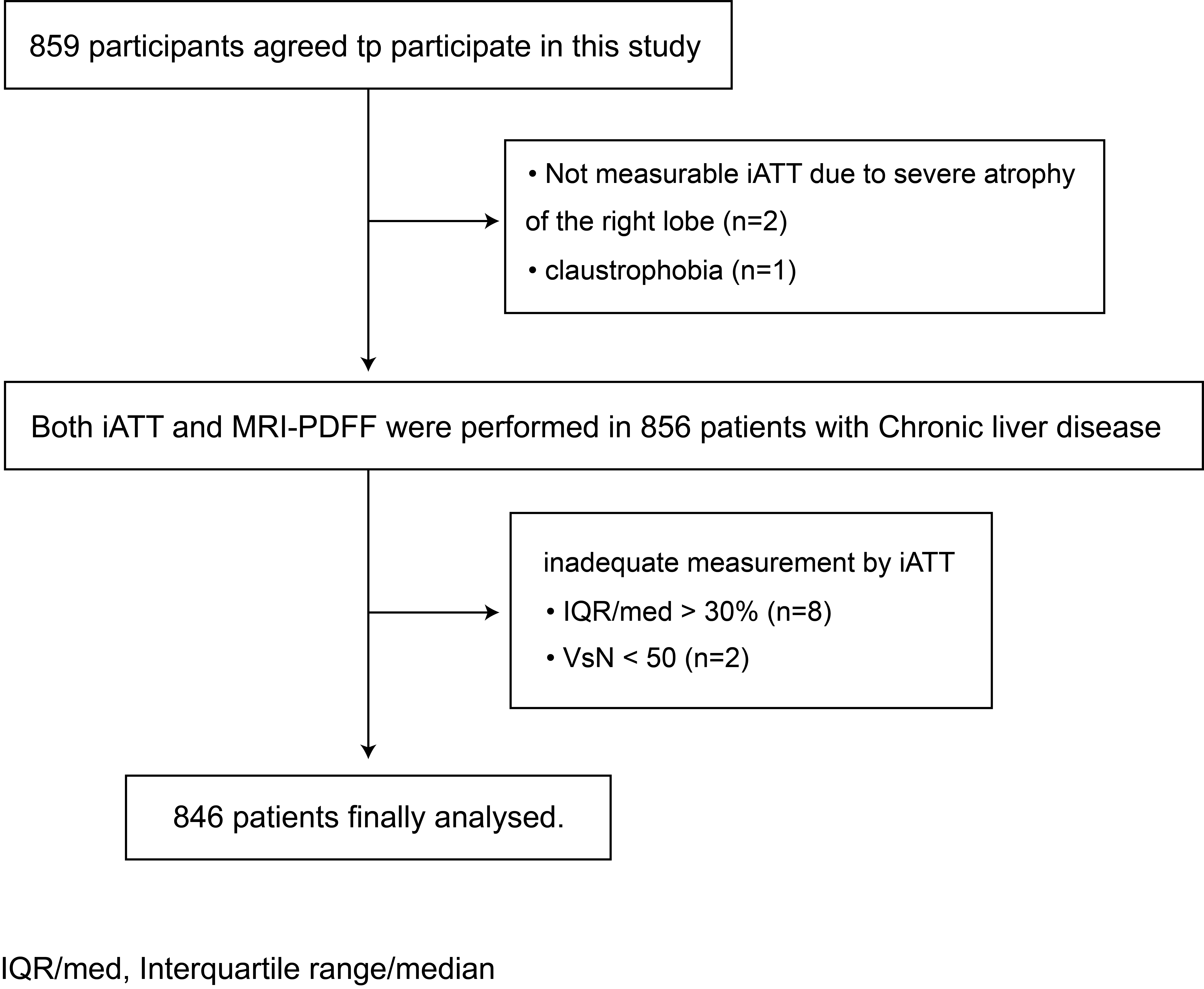

Supplement: Supplementary file 1 — Study design (TIF 4211 KB) [file 535_2024_2096_MOESM1_ESM.tif]

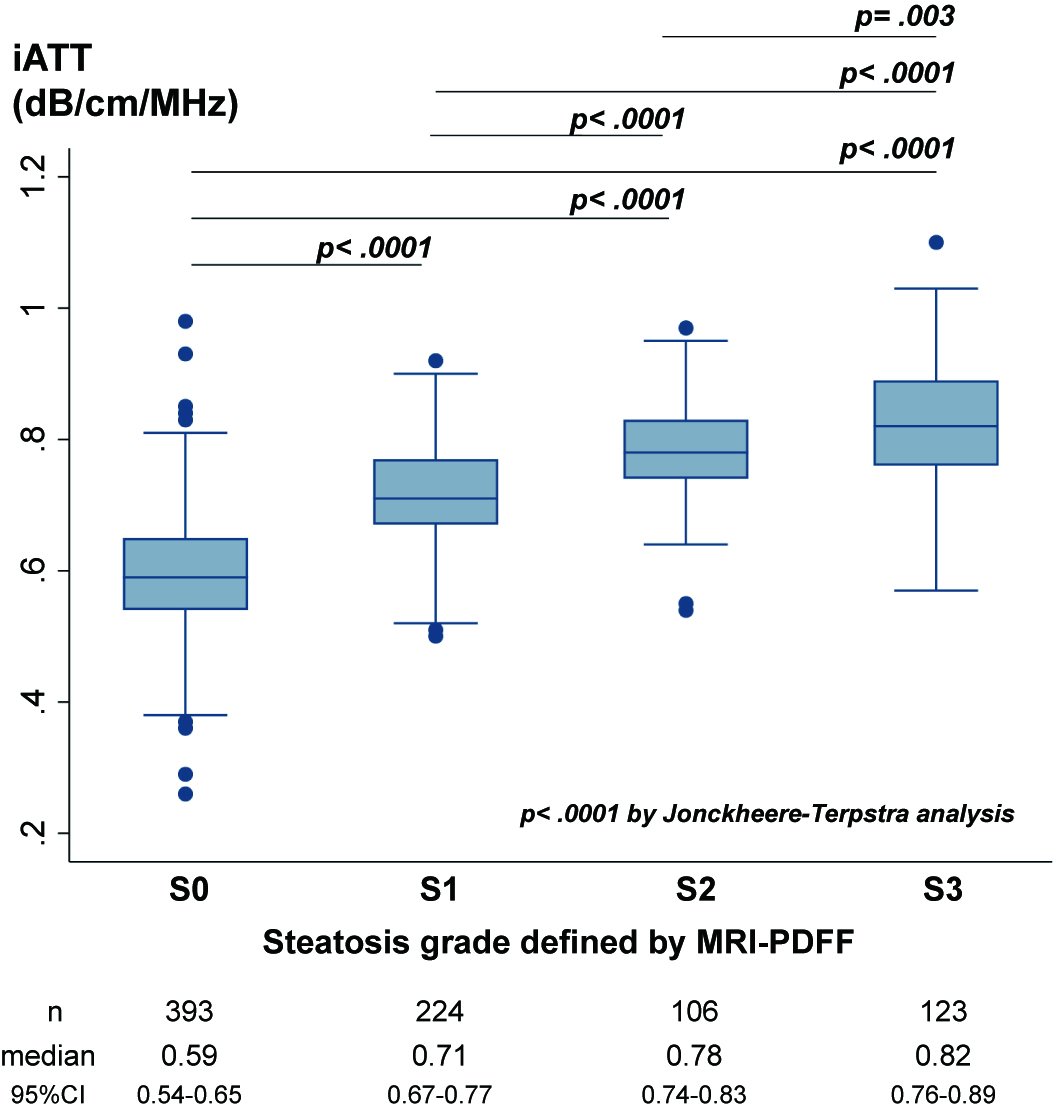

Supplement: Supplementary file 2 — iATT values according to steatosis grade. a grade 0, b grade 1, c grade 2, d grade 3 (JPG 476 KB) [file 535_2024_2096_MOESM2_ESM.jpg]

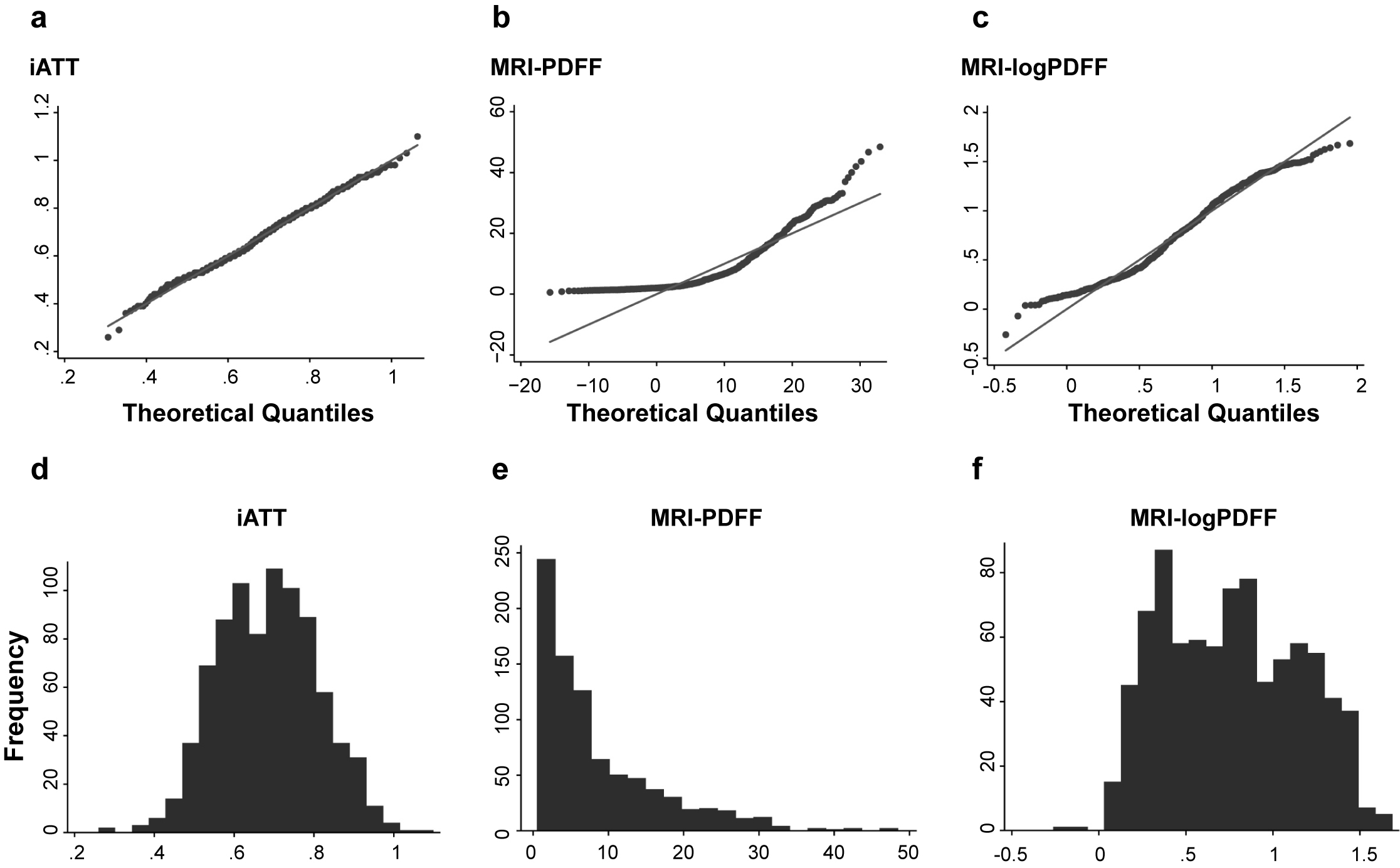

Supplement: Supplementary file 3 — Normal probability plot (a, d). iATT values were distributed normally, whereas MRI-PDFF values were not (b, e). MRI-PDFF values were log-transformed to have a normal distribution (MRI-logPDFF; c, f). MRI-PDFF, magnetic resonance imaging-proton density fat fraction (JPG 248 KB) [file 535_2024_2096_MOESM3_ESM.jpg]

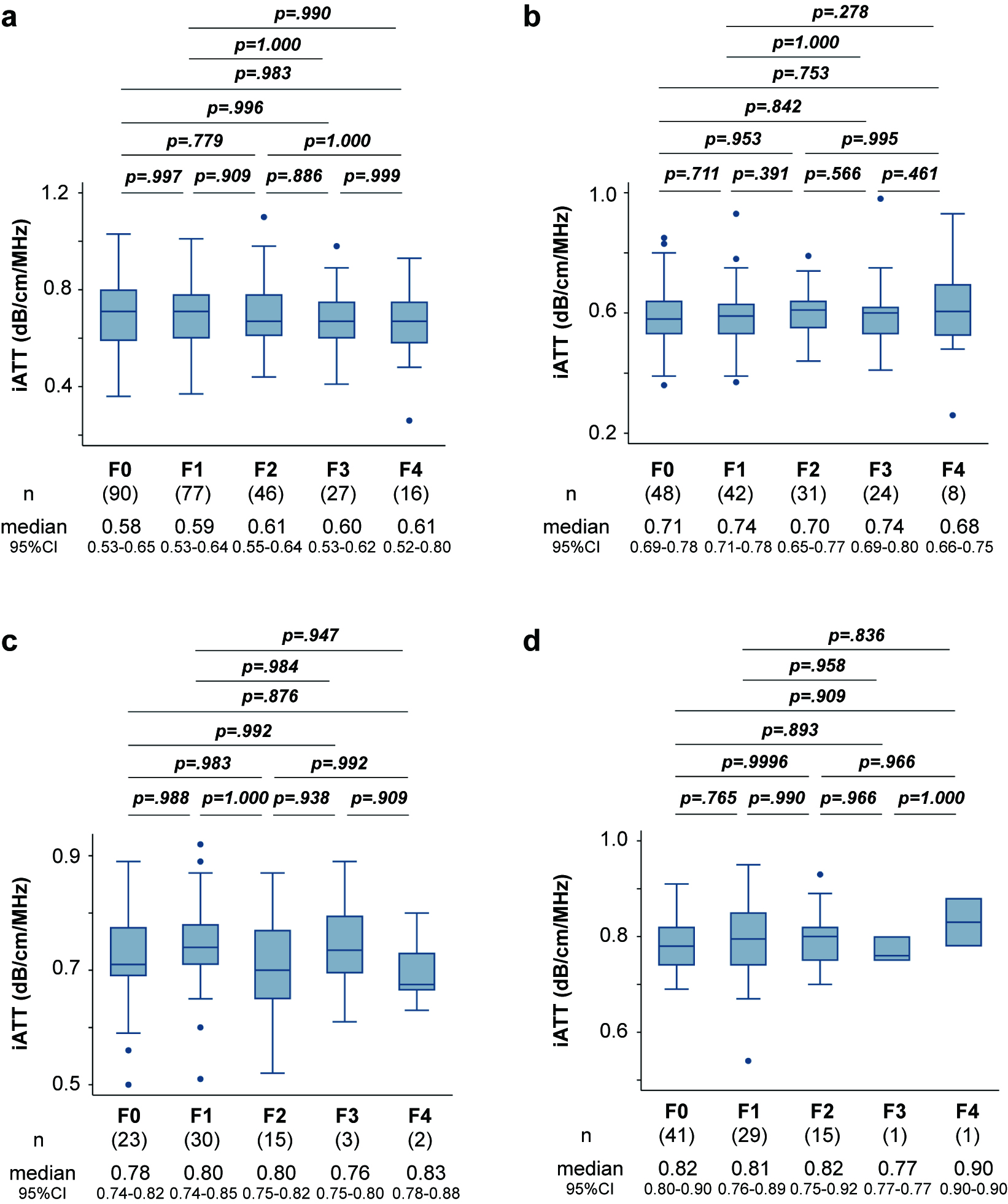

Supplement: Supplementary file 4 — iATT values according to fibrosis stage for steatosis grade subdivision. a S0, b S1, c S2, d S3 (JPG 1603 KB) [file 535_2024_2096_MOESM4_ESM.jpg]
